# Mobile X-ray outside the hospital: a scoping review

**DOI:** 10.1186/s12913-020-05564-0

**Published:** 2020-08-20

**Authors:** Maria Dietz Toppenberg, Thomas Erik Møller Christiansen, Finn Rasmussen, Camilla Palmhøj Nielsen, Else Marie Damsgaard

**Affiliations:** 1 The Department of Radiology, Aarhus University Hospital, Palle Juul Jensens Boulevard 99, 8200 Aarhus N, Denmark; 2 DEFACTUM, Social and Health Services and Labour Market, Olof Palmes Allé 15, 8200 Aarhus N, Denmark; 3 Department of Geriatrics, Aarhus University Hospital, Palle Juul Jensens Boulevard 99, 8200 Aarhus N, Denmark

**Keywords:** Mobile X-ray, Target population, Population health, Experience of care, Cost effectiveness

## Abstract

**Background:**

For several years mobile X-ray equipment has been routinely used for imaging in patients too unwell within the hospital, when transportation to the radiology department was inadvisable. Now, mobile X-ray examinations are also used outside the hospital. The literature describes that fragile patients may benefit from mobile X-ray, but we need to provide insights into the breadth, depth and gaps in a body of literature.

**Methods:**

The scoping review was performed by searching PubMed, Cinahl, Embase, EconLit and Health Technology Assessment.

English-, Danish-, Norwegian-, German-, Italian-, French- and Swedish-language studies, published 1.1.2009–1.5.2020 about mobile X-ray outside the hospital were included.

Participants were patients examined using mobile X-ray as the intervention.

PRISMA was used when eligible to build up the review. To extract data from the selected articles, we used a structured summary table.

**Results:**

We included 12 studies in this scoping review. The results were divided into four topics:1. Target population 2. Population health 3. Experience of care and 4. Cost effectiveness.

The main findings are that target population could be larger for instance including hospice patients for palliative care, group dwelling for people with intellectual disabilities, or psychiatric patients, population health may be improved, image quality seems to be good and mobile X-ray may be cost effective.

Limitations of language, databases and grey literature may have resulted in studies being missed.

**Conclusions:**

Mobile X-ray may be used outside hospital. There seems to be potential benefits to both patients and health care staff. Based on the published studies it is not possible to draw a final conclusion if mobile X-ray examination is a relevant diagnostic offer and for whom. Further studies are needed to assess the feasibility of use in fragile patients, also regarding staff, relatives and societal consequences and therefore the topic mobile X-ray needs more research.

## Synopsis

Questions:

Using mobile X-ray.

1. What are the target patient populations?
2. What are the improvements of population health?
3. What are the experiences of care?
4. Is mobile X-ray a cost-effective intervention compared to X-ray at hospital?

Findings: In this scoping review we found that the target populations in the studies were frail elderly, homeless, drug users, asylum seekers, patients with multiple co-morbidities, residents in care facilities outside a hospital setting, target screening for hard to reach populations and nursing home residents. The literature points at many potential outcomes, but without clear conclusions about the effect on population health, experiences of care, quality and cost effectiveness.

Meaning: In general, all studies fulfilled their aims, but claimed that further studies are needed to document the effect of mobile X-ray outside hospital.

## Background

### Rationale

For several years mobile X-ray has been used routinely for imaging patients too unwell to be transported to the radiology department for examination within the hospital for making diagnostic decisions [1]. Still it is used, when patients are too fragile to be transported to the radiology department [2–4]. Also mobile examinations have shown to be cost effective in the hard to reach populations for instance when screening for tuberculosis or in low or middle income countries [5–7].

In fragile patients e.g. nursing home residents, the environmental change from home to hospital for examination may result in cognitive difficulties [4, 8]. The patients experience disease deterioration, a need for increased care and medication for several days after the admission to the hospital [4, 8, 9]. In fragile patients, examination at the hospital can be a challenge due to transport to the hospital, long waiting times, and a need to be accompanied [10]. These patients also require extra care before, during and after the examination [10]. Image quality is a central aspect in X-ray imagining for correct diagnose of patients. A review published in 2017 [11] indicated that mobile X-ray for nursing home residents in high income countries is of comparable image quality to X-ray examinations at the hospital and have potential benefits, as mobile X-ray reduced transfers to and from hospital, increased the number of examinations carried out, and facilitated timely diagnosis and access to treatments. But they concluded that further research was needed to evaluate potential improvements in care quality and cost-effectiveness. Furthermore, the study population only included nursing home residents [11].

### Objectives

For reasons described above, mobile X-ray examinations are already used outside the hospital [12–14]. Our aim of this scoping review was to disclose published knowledge about the use of mobile X-ray and to provide insights into the breadth, depth and gaps in a body of literature.

For that reason, we asked four study questions:

Using mobile X-ray.

1. What is the target patient population?

2. What are the improvements of population health?

3. What are the experiences of care?

4. Is mobile X-ray a cost-effective intervention compared to X-ray at hospital?

## Methods

Protocol and registrations:

We used PICO (patient, intervention, comparison and outcome) and part of the PRISMA model to report the literature in this review studies [15]. This is because, this is a scoping reviews with the aim of disclosing published literature about the use of mobile X-ray and not a metaanalysis or effectiveness review. A protocol of the present study is available upon request.

### Inclusion criteria

Study design: Randomized controlled trials (RCT), non-randomized trials, cohort studies, case-control studies, cross – sectional studies, qualitative studies, case reports and series.

Countries: Australia, USA, Canada and Europe. We only considered these countries as comparable concerning X-ray equipment, patient facilities, transporting, environment, nursing staff and the purpose of using mobile X-ray.

Time period: 1.1.2009–1.5. 2020.

Language: Abstracts and/or articles published in the English, Danish, Norwegian, French, German, Italian and Swedish languages.

### Exclusion criteria

Study design: Ideas, editorials, personal opinions, letters, study plans, newspaper articles, protocols, posters, animal research studies, reviews and metaanalysis.

Intervention: Mobile X-ray used outside a hospital setting.

### Information sources

The following databases were searched: PubMed, Cinahl, Embase, EconLit and Health Technology Assessment. We chose these databases, because we considered that those databases would cover our study questions.

The search strategy and selection of databases were developed in cooperation with a librarian, expert in health-related literature search. The search strategy was developed in PubMed and was adapted to the other databases. In Table 1 the completed search strategy used is shown.

**Table 1:** Search strategy in PubMed, Cinahl and Embase for mobile X-ray

# Search number	PubMed
1	“Radiography” [Mesh]
2	“diagnostic” AND (x radiography* OR x ray* OR radiotherap*)* *
3	mobile AND (“radiography” OR x ray* OR radiotherapy*)**
4	transportable AND (“radiography” OR x ray OR radiotherapy*) **
5	Portable AND (“radiography” OR x ray* OR radiotherapy*)**
6	“X-rays” [Mesh]
7	“Nursing Homes” [Mesh]
8	“Homes for the Aged” [Mesh]
9	“nursing” AND (“home” OR “homes” OR facilit*)**
10	“home for the aged” OR “home for the elderly” OR “homes for the aged” OR “homes for the elderly”
11	((intermediate or “long-term”) AND care facility*) **
12	“hospital at home” **
13	“Mobile Health Units”[Mesh]
14	7 OR 8 OR 9 OR 10 OR 11 OR 12 OR 13
15	“Diagnostic Imaging” [Mesh:NoExp]
16	15 OR 1 OR 2 OR 3 OR 4 OR 5 OR 6
17	16 AND 14
18	17 NOT “mammography”
19	18 Filters: English; Danish, Norwegian; Swedish; German; Italian; French;

* meaning that the database searched for all words with different grammars

** non-MeSH

The search was carried out in December 2018, April 2019 and May 2020. If any new literature in the same search was published, the author received an e-mail. Supplementary search for image quality and cost effectiveness was carried out in May 2020.

In a search for image quality in December 2018, we identified 246 records, of which we ended up with 4 full text articles already found in the first literature search. The search strategy is shown in Table 2.

**Table 2:** Search strategy in PubMed for image quality

Search number	
1	“diagnostic” AND (“radiography” OR x ray* OR radiotherapy*)
2	mobile AND (“radiography” OR x ray* OR radiotherapy*)
3	transportable AND (“radiography” OR x ray* OR radiotherapy*)
4	portable AND (“radiography” OR x ray* OR radiotherapy*)
5	“Radiography” [Mesh] OR (#1) OR “x-rays” [Mesh]
6	Portable OR transportable OR mobile
7	#6 AND “Radiography” [Mesh] OR #1 OR “X-rays” [Mesh]
8	#1 OR #3 OR #4 AND #7
9	“diagnostic quality”
10	#quality”
11	“image quality”
12	#10 AND #8
13	“mammography” OR “ultrasound” OR “computered tomography” OR “Magnetic resonance” OR “Positron Emissions Tomography”
14	#12 NOT #13
15	#14 filters from publication date 2009/01/01
16	#15 filters English; Danish; Norwegian; Swedish

* meaning that the database searched for all words with different grammars

In a supplementary search in May 2020 in EconLit about mobile X-ray and cost effectiveness, we identified 12 records, of which no one could be included, because the literature did not fulfil our inclusion criteria. The search strategy is shown in Table 3. We also searched Health Technology Assessment using the same method as in Table 3, but we did not find any reports studying mobile X-ray.

**Table 3:** Search strategy in EconLit for mobile X-ray and cost effectiveness

Search number	
1	“diagnostic” AND (“radiography” OR x ray* OR radiotherapy*)
2	mobile AND (“radiography” OR x ray* OR radiotherapy*)
3	transportable AND (“radiography” OR x ray* OR radiotherapy*)
4	portable AND (“radiography” OR x ray* OR radiotherapy*)
5	# 1 OR # 2 OR #3 OR #4 AND cost effectiveness

* meaning that the database searched for all words with different grammars

### Search

#### Study selection

The records were archived and assessed using the computer program ‘Covidence’. In Covidence when screening the literature, in the selection you choose between ‘yes’, ‘no’ and ‘maybe’. All literature selected was double-checked by Co-author CPN. Reference lists in the included studies were screened and included if eligible and published within the time period.

#### Summary measures

To extract data from the selected articles, we were inspired by Peters to use a structured summary table for scoping reviews [16]. We included the following information for summarizing the data in the articles: Author and year, source origin, aim/purpose, study population, design/concept, intervention type, setting, organization, duration of the intervention, how outcomes are measured, key findings and limitations (see Table 3).

## Results

### Study selection

**Fig. 1:**
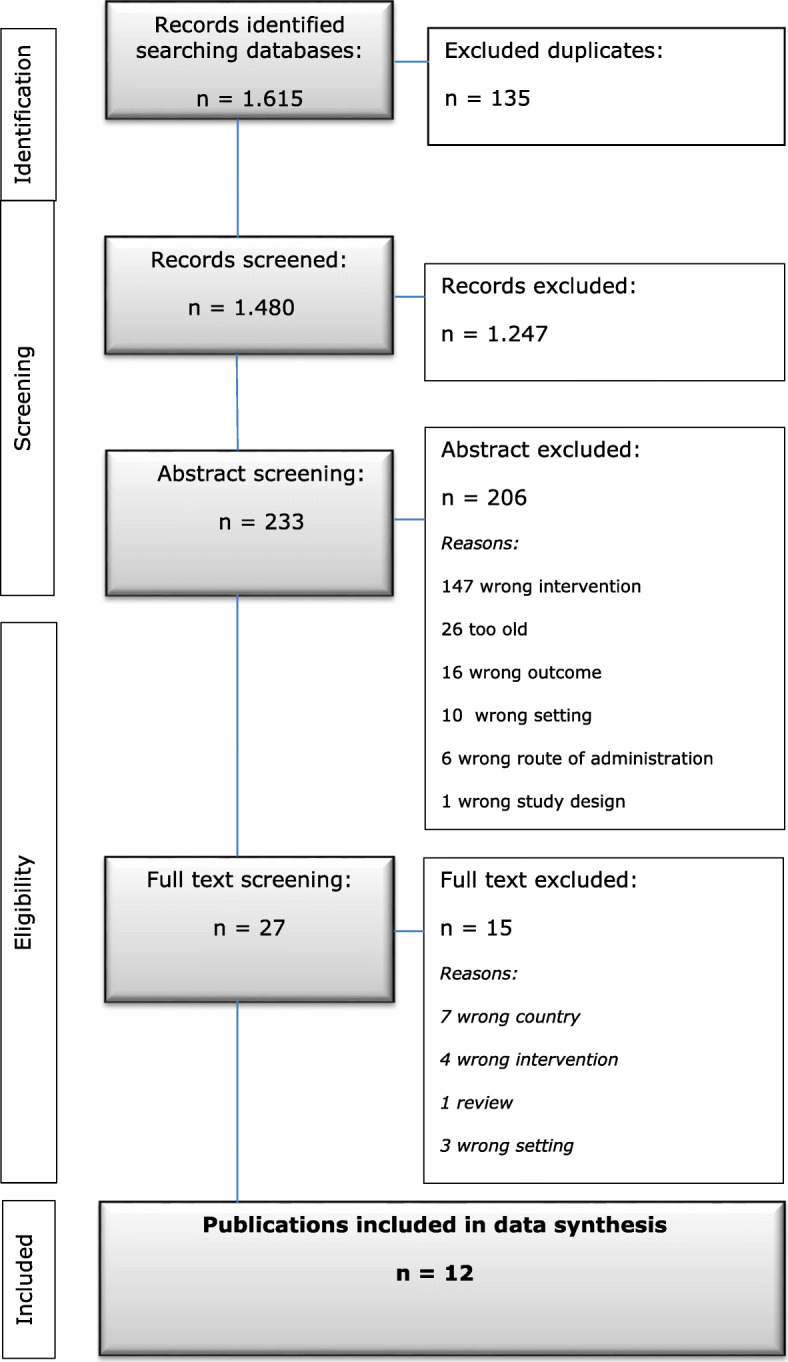
Flowchart of the selection process for literature search

In the literature search we identified 1.615 items. After removing duplicates, we had 1.480 records to appraise. Of these, 233 were selected for abstract screening after screening of titles. After reading the 233 abstracts, 27 full text articles were left to assess. In Fig. 1 an overview of the included and excluded studies and reasons for exclusion is presented from the searched in PubMed, Cinahl and Embase.

In Table 4 the data extraction of the 12 included studies is shown. One of the included studies was randomized [17], one study was cluster randomized [18] and one study was a randomized pilot study [19], while the rest is non-randomized or not ranging high in the evidence hierarchy. There was a variance in study design, power calculations and the number of patients (*n* = 69–1.192), but mobile X-ray was compared to hospital X-ray in all studies. The interventions were mobile X-ray and mobile X-ray combined with hospital X-ray [17–24, 26–29]. The most common X-ray examinations were of chest [17, 20–22, 26–29], hip and pelvis spine [17, 20–22, 26–29] and abdomen [17, 20–22, 26–29]. Some studies only included chest X-rays [19, 21].

**Table 4:** show the results of the included literature

Author and year	Source origin	Aim/Purpose	Study population	Design/Concept	Intervention type	Setting	Organization	Duration of the intervention	How outcomes are measured	Key findings	Limitations
Precht 2019 [17]	Denmark	To compare image quality of chest, hip and pelvis images taken at the mobile X-ray equipment in nursing homes.	Examinations of chest (*n* = 20), hip (*n* = 64), and pelvis (*n* = 32) equally obtained from each setting of mobile X-ray (ME) and stationary equipment (SE) from patients 70+ years.	RCT.	Mobile X-ray.	Nursing homes and hospital.	Hospital.	January 2018 -?	The images were viewed separately and scored according to Visual Grading Analysis (VGA).	- The VGA showed higher image quality for SE system while the contrast-Detail Radiography phantom showed higher image quality for the ME system.	- The reporting radiographers could recognize some of the images from ME examinations even though the images were blinded.
Aldridge 2012 [18]	England.	To compare engagement strategy for mobile TB screening	Homeless people in 59 hostels (*n* = 1.192).	- Cluster randomized. - Quantitative.	Mobile X-ray.	Hostels for the homeless in London.	A National Health Service, ‘Find and Treat’ led the mobile X-ray service.	Feb. 2012 to Oct. 2013.	The number of eligible clients at a hostel venue screened for active pulmonary tuberculosis by the mobile X-ray *n* = 2.342.	- Of 59 eligible hostels, 46 were randomized. - Paper includes number in control *n* = 1.192 eligible with median 45% uptake Intervention *n* = 1.150 with median 40% uptake	- Lack of individual data - Power calculation was performed but not at person level.
Ricauda 2011 [19]	Italy.	To explore the quality of imaging and clinical outcomes using mobile X-ray.	Frail elderly patients already attending Hospital at Home Service (*n* = 69).	- RCT as a part of a pilot study. - Quantitative.	Mobile X-ray vs. X-ray at the hospital.	At the patients own home.	I in corporation with the hospital.	June 2008 to June 2009.	- Confusion Assessment Method (score). - Satisfaction.- Image quality.	- After X-ray examination an acute confusional state requiring treatment occurred in 17% of the patients in the hospital group vs. 0% in the mobile X-ray group. - 94% of patients examined with mobile X-ray were satisfied. - No differences in image quality.	- The study is a pilot study - There was no sample size calculation. - No description of the satisfaction measures. - Patients who needed an urgent examination (within 24 h) and patients needing an X-ray examination not suitable at home were excluded. - Only patients who are referred to X-ray examination of thorax is included
Dozet 2016 [20]	Sweden.	To determine whether examinations of patients in elderly care facilities with mobile radiography were cost effective from a societal perspective compared with hospital based radiology examinations.	Nursing home residents in two different areas (*n* = 312).	- A prospective study. - Quantitative.	Mobile X-ray.	10 Nursing homes.	Corporation with the hospital radiograph service.	Nov. 2012 to May 2014.	Questionnaires distributed to the nursing homes . Mobile X-ray (*n* = 312). X-ray at the hospital (*n* = 71)	- Mobile X-ray has significant lower costs per examination compared with hospital based radiography. - Differences in health care related costs were also significant lower using mobile X-ray.	- The study only measured health care related costs. - An imbalance in number of participants from the two study groups. - Participation was voluntary so not all patients replied on the questionnaire. -no response rate
Eklund 2012 [21]	Sweden.	To investigate the usefulness of a mobile radiography service for radiological assessment of patients in nursing homes from the patient and staff perspectives.	Nursing homes patients.	- Feasibility study where patients (*n* = 123) and staff (*n* = 123) answered questionnaires. - Quantitative.	Mobile X-ray services for nursing home residents.	10 Nursing homes	Part of the hospital service.	Sep. 2008 to Sep. 2009	Questionnaires measuring patients and staff experience with the mobile X-ray service.	- Security and comfort. - Acceptance from the patients. -No need for transportation. -No need for staff to be absent from the nursing home.	- Data before mobile X-ray are based on estimates on time from the healthcare staff. - Out of 123 patients 62 were able to answer the questionnaire about patient satisfaction. - The questionnaire is not published. - The image quality is not directly being measured. - A small study population.
Montalto 2015 [22]	Australia.	1. To describe the activity of the mobile X-ray service (MXS), its recipients and the number and type of plain X-rays performed. 2. to measure the impact of the mobile X-ray service on the emergency department attendances by residents of residents of the residential aged care facilities (RACF) who require plain X-ray services.	The top 30 RACF users of mobile X-ray service in Melbourne (*n* = 919).	- Descriptive study, that uses before and after cohort approach. - Quantitative.	Mobile X-ray vs. X-ray at the hospital.	The Mobile X-ray Service was offered in the northern and western regions of Melbourne to nursing home residents.	Organized from the hospital.	1 July 2012 to 30 June 2013.	All plain X-rays requested by and/or conducted on residents from the 30 RACF.	- The MXS delivered 1.532 services attendances to 109 different RACFs. - Most patients were bed or wheelchair bound followed by those who needed assistance to ambulant. - There were an 11,5% reduction in Emergency Department representation the year where mobile X-ray was offered.	- The study was conducted during implementation of MXS. - Based on a single service on a single hospital. - The study is not randomized. - The study population was chosen from the authors based on their use of MXS. - There was no patient payment as in other services. - Some of the authors were involved in the service delivery.
Story 2012 [23]	England.	To establish the sensitivity and specificity of mobile digital CXR and to test the hypothesis that actively identified cases have reduced the odds of sputum smear positivity vs. those presenting passively to health care services from the same populations.	Homeless, drug users and asylum seekers (*n* = 352).	- Observational study. - Quantitative.	Screening using mobile X-ray.	Homeless hostels, day centers, drug treatment services and prisons in London.	Part of the Hospital service.	April 2005 to March 2010.	All individuals were included, sensitivity and specificity was calculated	- The intervention had a sensitivity of 81% and a specify of 99%. - Cases identified through screening were less likely to be smear-positive than passively identified cases.	- Small patient group. - Analysis is based on existing data, meaning that confounding variables was not possible. - The time period when data was collected vary and may impact the result.
Thingnes 2010 [24]	Norway.	To explore knowledge about expectations, meanings and opinions concerning implementing mobile X-ray at nursing homes.	Nursing home residents.	- Focus group interview with an unknown number of participants in the three groups: Nurses, health care staff and radiographs.- Qualitative.	Mobile X-ray.	Nursing home already included in a pilot project.	Mobile X-ray is organized from the hospital.	2 months, intervention.	Transcription and recording interviews.	- Everyone thought that mobile X-ray would be a great advantage for the patients due to no transportations to the hospital. - Implementation of mobile X-ray demand great corporation between healthcare staff, great communication and maybe an increased workload.	- The study is based on expectations and not on experiences. - The interview did not include doctors and secretaries, which means that we do not get their point of view.
Kjelle 2018 [25]	Norway.	To analyze the cost of with a social perspective of X-ray examination and treatment of nursing home residents.	Simulation of nursing home residents (*n* = 1.000).	- A case control study.- Quantitative.	Two alternatives were compared, including a hospital-based service and a combination of hospital-based and mobile radiography.	Hospital compared to nursing homes.	Mobile X-ray was offered from the Department of Radiology at the hospital.	Data was collected in 2015.	Costs based on the 2016 Norwegian kroner converted to the Euro.	- Cost per examination at the hospital was EUR 2.790 and in combination with mobile X-ray and hospital EUR 1.946.	- Effects of mobile X-ray service were not evaluated only costs. - When real data could not be found, assumptions were made. - Cost of treatment and ambulance transportation do not have high influence of the result.
Kjelle 2019 [26]	Norway	To determine the utilization of diagnostic imaging among nursing home residents and if there were differences between hospitals with or without mobile X-ray service.	11.066 examinations of nursing home residents.	- Data from radiological information systems of 11 hospitals. -Quantitative.	Mobile X-ray.	Nursing homes and hospital.	Hospital.	All diagnostic imagine procedures for nursing home residents in year 2015.	Data were collected from the radiology information systems, of 12 different hospitals.	- Mobile radiography services increase the level closer to the user rate in the general population.	- The study did not compare before and after implementation data, but compared hospitals with mobile X-ray service to hospitals without mobile X-ray service.
Vigeland 2017 [27]	Norway.	To examine the use and benefit of a mobile X-ray service that enables imagining at nursing homes.	The study population is nursing home residents. Questionnaires on behalf of the patients fulfilled by: referring doctors (*n* = 300) and follow-up doctors (*n* = 100).	- Cohort study based on a pilot study. - Quantitative.	Satisfaction with mobile X-ray.	42 Nursing- and assisted living homes in 10 municipalities in Norway.	Oslo University Hospital was responsible for the mobile X-ray service.	March to Sep. 2015.	Questionnaires to referring doctors and follow-up doctors.	- In 73% the patients would have been sent to the hospital radiology department if the mobile X-ray service had been available. - In 20% the patients would not have been examined.	- Is a part of a pilot project. - The questionnaires are only for doctors. - The response rate is low. - There are no end points measured. - No data before and after implementing mobile X-ray for instance concerning hospitalization.
Kjelle 2018 [28]	Norway	To explore success criteria and barriers in the process of implementing mobile radiography service from the point of view of the hospital and municipal managers.	Information from managers from five hospitals and six municipalities.	- Interview study. - Qualitative.	Mobile X-ray.	Hospital and municipality where mobile X-ray had been implemented.	Hospital.	Feb 2016 – may 2016.	Interviews.	- Financial, structural and procedural barriers. - Main success criteria were external funding and support and engagement	- Recruitment was based on the organizations finding a volunteer to represent them in the interview.

What is the target patient population?

As shown in Table 4 the study populations in the included literature were frail elderly [19, 22], dementia patients [19], homeless [18, 23], drug users [23], asylum seekers [23], and nursing home residents [17, 20–22, 26–29].

What are the improvements of population health?

Improvements of population health are measured by several different outcomes that by proxy may indicate if health status is improved. The outcomes of the 12 studies were delirium measured by confusion assessment method [19], sensitivity and specificity of mobile X-ray to find tuberculosis [18], patient and health care satisfaction measured by qualitative interviews [24, 28] and questionnaires [19–21, 27], image quality and costs [18–28].

In one study the authors suggest that mobile X-ray seems to increase the certainty of presumed diagnoses, so that treatment could be avoided in many cases [27]. Examination using mobile X-ray could also prevent patients from being treated at the hospital [22]. Fewer patients may need transportation to the hospital, and probably fewer patients would become delirious [19, 21, 22, 27]. The literature also describes places to use mobile X-ray outside the hospital for instance in nursing homes [17, 20–22] and shelters [18, 23].

For nursing home residents mobile X-ray was considered a reasonable alternative to hospital X-ray examination, because they could be treated at home [21]. Treating patients at home reduced the incidence of delirium [19]. Less transfer to the hospital was a positive outcome, since transportation of patients from their homes to the hospital may worsen the condition of demented or disorientated patients [19, 21, 22, 27]. Examination in the familiar surroundings may calm the patients, as insecurity during transportation to hospital is experienced as pain or confusion [19, 22, 23, 25, 27, 28].

What are the experiences of care?

The five included studies explored the quality, usefulness, knowledge, barriers, success criteria’s and expectations of mobile X-ray offered to nursing home residents [17, 21, 24, 27, 28]. In 5 studies patients, healthcare staff, nurses and referring doctors were asked using both qualitative [24, 28] and quantitative methods [17, 21, 27]. The literature found that the main part of patients and health care staff was satisfied with mobile X-ray examination and the benefits that mobile X-ray had for both patients and staff [19, 21, 24–28]. Results showed high patient acceptance of mobile X-ray as the patients were happy not having to go away for several hours, felt safe and that it was much better than going to the hospital for examination [21, 24, 28]. In none of the studies the patients had a negative opinion of the procedure. Nursing home staffs pointed out beneficial factors such as the security and comfort for the patients, who could remain in their usual environment, no need for transportation, and no need for staff to be absent from the nursing homes while accompanying the patient to the hospital [19, 21, 27]. Barriers to implement mobile X-ray were identified as organizational changes, financial barriers and structural changes for the staff [28]. Thus implementing mobile X-ray needs good relations between the nursing home and the organization providing mobile X-ray [28].

Several studies point out that the diagnostic quality of the images may be a challenge, since the health care staff may have to choose between good enough image quality with no transportation of patients and optimal image quality with transport [19, 21, 22, 24, 25]. Prech et al. studied image quality of chest, hip and pelvis images using Visual Grading Analysis and found that there were no significant differences in image quality between mobile X-ray and X-ray at the hospital [17]. Kjelle et al. studied the utilization of diagnostic imaging among nursing home residents and if there were differences between hospitals with and without mobile service. The authors found a lower use of more advanced radiology by nursing home residents compared to the general population and indicated that mobile X-ray resulted in fewer CT and ultrasound examinations at hospital [25].

Is mobile X-ray a cost effective intervention compared to X-ray at the hospital?

We found one prospective study comparing costs between mobile X-ray and X-ray at the hospital [29]. The authors found significant differences in costs between mobile X-ray and X-ray at the hospital from a societal perspective. The societal benefit to the elderly patient at nursing homes was high, reducing the anxiety and possible risks associated with transfer from the nursing home to hospital for radiography [20].

Kjelle et al. described in their interview study that it was important to get support from the top management in all organizations, which was a challenge [28]. The support was necessary in order to get money allocated to the project. Financial barriers would result in managers at the hospitals not being willing to invest in mobile equipment, staff and vehicle. Even though mobile X-ray may save money, because of fewer hospitalizations and less transporting the savings are not always visible in the department budget at the hospital [25].

Overall the literature suggests that mobile X-ray is cost effective compared to X-ray at the hospital, but this is not supported by evidence from a RCT. The studies investigate costs such as cost per patient, salary, capital costs of equipment and operating costs [21, 24, 25]. Many patients would not be examined, had mobile X-ray service not existed [27].

## Discussion

### Summary of evidence

The purpose of this scoping review was to identify published knowledge about mobile X-ray examination outside the hospital compared to examination at the hospital in high income countries from 2009 till now. Other reviews included a specific target population or outcome measure in their studies, but by including all type of patients and outcomes, we hoped to find results that could show which study design and outcome measures should be used to document the effect of mobile X-ray. By including all literature several different qualitative and quantitative methods were described to measure outcomes such as population health, experience of care, quality and costs. Also the quality of the studies differed a lot and there was no agreement about the appropriate outcome measures. It was surprising that only 12 studies could be included in the review, but when reading the studies, we found that mobile X-ray is a difficult topic with many aspects to consider, when defining target population and measuring effects such as population health, experience of care and costs.

### Target population

We found that the target population was frail elderly, demented patients, homeless, drug users, asylum seekers and nursing home residents [18–24, 26–29].

Other patient groups may also be included or at least studied as possible target populations, e.g. hospice patients for palliative care, group dwelling for people with intellectual disabilities, or psychiatric patients. In defining the target population country, environment and specific factors may also influence the definition of the relevant target population. The problem is also, that the target population might differ in each country and therefore it may not be possible to define a specific target population for mobile X-ray in general.

To define specific outcomes of mobile X-ray, a specific target population and location is needed. Mobile X-ray could be used in other locations than described in the literature, e.g. at the local general practitioner (GP), in a healthcare centre in order to meet the ambulant patient’s needs, but also the needs of the health care staffs, crowded hospitals and general practitioners. We do not know if the locations described are the adequate locations, since it may differ in each country [18–25, 27].

### Improvements of population health

We found that improvements of population health were: increasing the certainty of presumed diagnoses, so treatment could be avoided in many cases [22], prevent patients from being treated at the hospital [20], fewer patients may need transportation to the hospital [21, 24, 27], and probably fewer patients would become delirious [19].

The measurements for improved population health are not clear, for instance consequences of transportation, environmental changes or waiting time for the patient. Another problem is measuring the effect of mobile X-ray all studies conclude that further studies are needed to measure the effect, but at the same time they found that mobile X-ray probably is beneficial to the patient in different ways. The problem is that one outcome measure may be relevant for one patient group but not for all patient groups. For demented patients delirium may be a relevant outcome measure, for a homeless, sensitivity and specificity of detecting tuberculosis may be more relevant. The outcomes of the studies describing improved population health give a mixed and unclear indication of what to be used as outcome measures and study design [18–24, 26–29].

### Experiences of care

Experience of care was mostly measured as satisfaction and we found that the included patient and healthcare staff seemed to be satisfied with mobile X-ray [19, 21, 24]. Also the image quality is good [17, 19]. But the question is, if satisfaction is directly comparable to experience of care.

In all studies we only found positive results. But experience of care and satisfaction may not be comparable between different patient populations and different health care staffs. When asking a demented nursing home resident, relatives or health care staff about their satisfaction with mobile X-ray, no transportation or preventing the possible effects of delirium could be related to high satisfaction [19, 21, 24]. Asking homeless residents or asylum seekers about satisfaction, these outcome measures probably would not even be relevant. Therefore studying experience of care it is necessary to be very specific of study group and aim. It could be relevant to ask the patients about their experiences being examined with mobile X-ray, but it may be difficult with certain patient groups, e.g. patients with severe dementia.

The literature shows that mobile X-ray may facilitate high quality of treatment and care [17, 19–21, 24, 27]. The question is, if the quality of the studies permits making conclusions concerning experience of care, since we did not find two studies measuring experience of care, using the same outcome in an identical population. We find, that the target population for measuring experience of care could also be other groups than the patients and health care staff in the studies. For instance GP, heads of departments, relatives or other persons involved in mobile X-ray, who could express their satisfaction. When asking the referring doctors if the mobile X-ray examination had given important information to patients and their families, they replied positively [19, 21, 25, 27]. In the qualitative study by Kjelle, the authors found that the general quality of care in nursing homes was considered to be improved, because healthcare staff did not have to arrange for volunteers or family to accompany patients to the hospital or the staff had to accompany the patients [28].

The literature shows that measuring experience of care is difficult [19, 21, 27, 29] and it may be the reason why, no one has documented a gold standard for doing that. This is probably because the patients are hard to reach and therefore, they might have difficulties sharing their experiences of mobile X-ray [20]. Information from referring doctors, healthcare staff, and relatives may be biased and not representing patients’ views. The image quality is good and reduces the use of other imaging examinations in nursing home residents [17]. The image quality seems to be good and while conducting this scoping review new studies concerning image quality have been published, so the quality is in focus [17].

### Cost-effectiveness

Mobile X-ray seemed in one study to be cost effective, but using costs as an outcome measure, all relevant costs of mobile X-ray must be considered and compared to X-ray at the hospital to conclude if mobile X-ray is cost efficient [20, 29]. It is suggested that probably the costs are lower using mobile X-ray seen in a social perspective not including derived costs for instance costs for relatives accompanying the patient [29].

### Limitations

There are several limitations of our scoping review. Limiting the review by language, years and locations may have resulted in key studies being missed. However, we wanted to look at literature describing mobile X-ray in a western context within the last years. On the other hand mobile X-ray is being used in India, so it could be relevant to include a broad literature in a systematic review. Choosing only to search few economic specific databases could also have limited the findings, but we find that all literature about mobile X-ray would be published in healthcare journals, since we did not find any reports in the economic databases.

## Conclusions

In conclusion, this scoping review indicates that mobile X-ray in high income countries may be used outside the hospital in nursing homes, homes for the elderly and in shelters. Patients and health care staff seem to be satisfied with mobile X-ray. The image quality is good and mobile X-ray may be cost effective. In general, the included literature may lack the evidence for documenting the effect of mobile X-ray, maybe because the effect is difficult to measure in a broad population. There are challenges documenting the effect of mobile X-ray yet, mobile X-ray has come to stay even if we still need a clear answer on how to develop the mobile X-ray, to whom it should be offered and therefore the topic needs more research.

### Future research

Mobile X-ray is a relatively unexplored and new field and therefore much research needs to be conducted. Future research could for instance be RCT measuring the effect, finding target populations, patient satisfaction and/or cost effectiveness.

## Data Availability

“All data generated or analysed during this study are included in this published article”.

## Abbreviations

CT: Computered tomography
GP: General practitioner
MXS: Mobile X-ray service
RCT: Randomized controlled trial
RACF: Residential aged care facilities
PICO: Patient, intervention, comparison and outcome
